# In vitro bioavailability‐based assessment of the contribution of wild fruits and vegetables to household dietary iron requirements among rural households in a developing country setting: The case of Acholi Subregion of Uganda

**DOI:** 10.1002/fsn3.1977

**Published:** 2021-01-07

**Authors:** Jean Damascene Tuyizere, Lawrence Okidi, Samuel Elolu, Duncan Ongeng

**Affiliations:** ^1^ Faculty of Agriculture and Environment Department of Food Science and Postharvest Technology Gulu University Gulu Uganda

**Keywords:** antinutrients, household annual dietary iron requirements, iron bioavailability, wild fruits and vegetables

## Abstract

Wild fruits and vegetables (WFV) are believed to contain substantial quantities of micronutrients and are commonly consumed in rural areas of developing countries endowed with natural vegetation. Previous studies that provided evidence on the contribution of WFV to household micronutrient intake in a developing country setting did not consider the effect of antinutritional factors. Therefore, applying the in vitro bioavailability assessment technique and using the Acholi subregion of Uganda a case area, this study examined the contribution of commonly consumed WFV to the pooled annual household dietary requirement for iron. Laboratory analysis showed that the concentration of antinutrients varied with plant species but the pool was dominated by phytate (10.5–150 mg/100 g) and phenolic substances (38.6–41.7 mg GAE/g). In vitro iron bioavailability varied with plant species was quantitatively higher from vegetables than fruits by 27% although total concentration of the micronutrient was higher in fruits than vegetables by 142%. Nutritional computation, taking into account, household composition, and physiological status revealed that consumption of WFV resulted in a median contribution of 1.8% (a minimum of 0.02 and a maximum of 34.7%) to the pooled annual household dietary iron requirements on the basis of bioavailable iron fraction. These results demonstrate that WFV contributes meagerly to household iron needs but may serve other dietary and non‐nutrient health purposes.

## Graphical abstract

The contribution of wild fruits and vegetables to household micronutrient needs has not been adequately quantified in a developing country setting. Using Achali subregion of Uganda as a case area, and applying the in vitro bioavailability assessment technique, nutritional computation of household nutrient needs revealed that consumption of wild food plants contributes meagerly to household dietary iron needs.

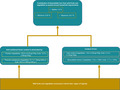

## 1 INTRODUCTION

Hunger remains a major global development challenge as articulated under the 2030 sustainable development goals agenda of the United Nations (SDG number 2) (UN, 2015a). It is estimated that 9.3% of the global population (689 million people) suffer from chronic hunger with the highest prevalence occurring in food insecure developing regions of the world such as East Asia and Sub‐Saharan Africa (SSA) (FAO et al., 2017; FAO et al., 2015; Rosen et al., 2016; Godfray et al., 2010; FAO, 2019). Investment in actions that improve food security situation has been recognized as effective strategies to ameliorate hunger (Godfray et al., 2010), and a number of positive outcomes have been achieved over the last decades. A typical example is the reduction in the prevalence of food insecurity from 32.8% to 26.8% observed in SSA between 1990 and 2012 (FAO et al., 2012; Fraval et al., 2019). Despite this achievement, SSA remains the region with the highest proportion of hunger affected population globally (1 in every 5 people) (FAO, 2019).

In SSA and other developing regions of the world, food insecurity is highly associated with both macro‐ and micronutrient undernutrition (Engelbert et al., 2013; Xie et al., 2018). Among micronutrients, iron deficiency remains the most common nutritional challenge of public health importance with 43% of children under 5 years and 29% of women of reproductive age being iron deficient (Stevens et al., 2012; Baker & Greer, 2010; Dupont, 2017; Sharma & Dhandoria, 2019). Besides causing developmental consequences (e.g., stunting, impaired cognitive development, increased childhood, and maternal morbidity), iron deficiency in the context of nutrition transition may present additional deleterious health consequences when combined with obesity and other related noncommunicable chronic diseases (Eckhardt, 2006; Nazia et al., 2019).

Despite the well‐known negative health consequences associated with inadequate iron availability in the human body, intake of iron is one of the lowest among essential micronutrients in developing countries. This situation predisposes vulnerable human groups (pregnant women, children, adolescents, and the elderly) in such countries to iron deficiency health complications (Loh & Khor, 2010; Means, 2020). For instance, in Uganda, 53% of children under five are iron deficient, and 32% of women are anemic, while iron deficiency anemia accounts for 75% of all anemia during pregnancy (UBOS, 2017; Horowitz et al., 2013). Low intake of micronutrients such as iron has been found to be critical most especially among the poor segments of the population that live in rural areas in developing countries (Angeles‐Agdeppa et al., 2019; Biesalski & Black, 2016). This is notwithstanding the fact that the majority (62%) of the population in such countries live in rural areas (World Bank, 2018) and lack financial resources to access highly bioavailable iron‐rich animal source foods (Aguilar & Sumner, 2020).

Wild fruits and vegetables (WFV) are still abundantly present in rural areas of many countries in developing parts of the world (Ahmed, 2019; Bvenura & Sivakumar, 2017) and an estimated one billion people, especially those in SSA include edible WFV in their diets (Bharucha & Pretty, 2010; Cordeiro, 2012; Shumsky, 2012; Sunderland & Rowland, 2019). Uganda ranks high among African countries where households utilize WFV for human nutrition (Ojelel et al., 2019; Smith & Ezyaguirre, 2007), and the Acholi and Karamoja subregions in particular are well‐known for that nutrition behavior (Loki & Ndyomugyenyi, 2016; Okidi et al., 2018; Okori et al., 2009; Oryema et al., 2013). Consumption of WFV in these regions is largely guided by traditional ecological knowledge (Arenas & Scarpa, 2007; Pardo‐de‐Santayana et al., 2005). Economically, the significance of WFV on household nutrition lies on the fact that they are regarded as low‐input, low‐cost option for improving household nutrition and reducing the need to spend limited financial resources during incidences of crop failure, drought, or conflict‐driven famine (Fentahun & Hager, 2009; Gordon & Enfors, 2008; Jama et al., 2008; Asprilla‐Perea & Diaz‐Puente, 2019).

Information available in literature indicates that WFV, in areas where they are consumed, contribute significantly to household intake of essential micronutrients such as iron (Borelli et al., 2020; Okidi et al., 2018). This is because wild plant species have been found to be richer in micronutrients compared to the cultivated variants (Mavengahama et al., 2013). Nonetheless, the occurrence of unacceptably high prevalence of anemia among children under five (71%) and women of childbearing age (47%) in certain areas such as the Acholi subregion (UBOS, 2017) raises question on the effective contribution of WFV to household nutritional iron needs in areas where they are consumed. Previous studies that assessed the contribution of WFV to household nutrition in Acholi subregion focused on total iron without due attention to bioavailability of the nutrient (Okidi et al., 2018) and yet plant species in general contain antinutritional factors such as phytates, oxalate, tannins, and saponin that potentially reduce iron bioavailability from plant‐based foods (Mihrete, 2019; Natesh et al., 2017) thereby exposing resource constrained rural communities that rely on plant‐based foods to high risk of iron deficiency and related health complications. Using the Acholi subregion of Uganda as a microcosm for rural areas in developing countries where WFV are consumed and applying the in vitro bioavailability‐based technique and household nutritional needs computation, this study examined the contribution of WFV to household requirement for bioavailable iron. This information is necessary for planning nutritional interventions to improve micronutrient intake in areas where WFV are consumed.

## 2 MATERIALS AND METHODS

### 2.1 Study design and study area

**Figure 1:**
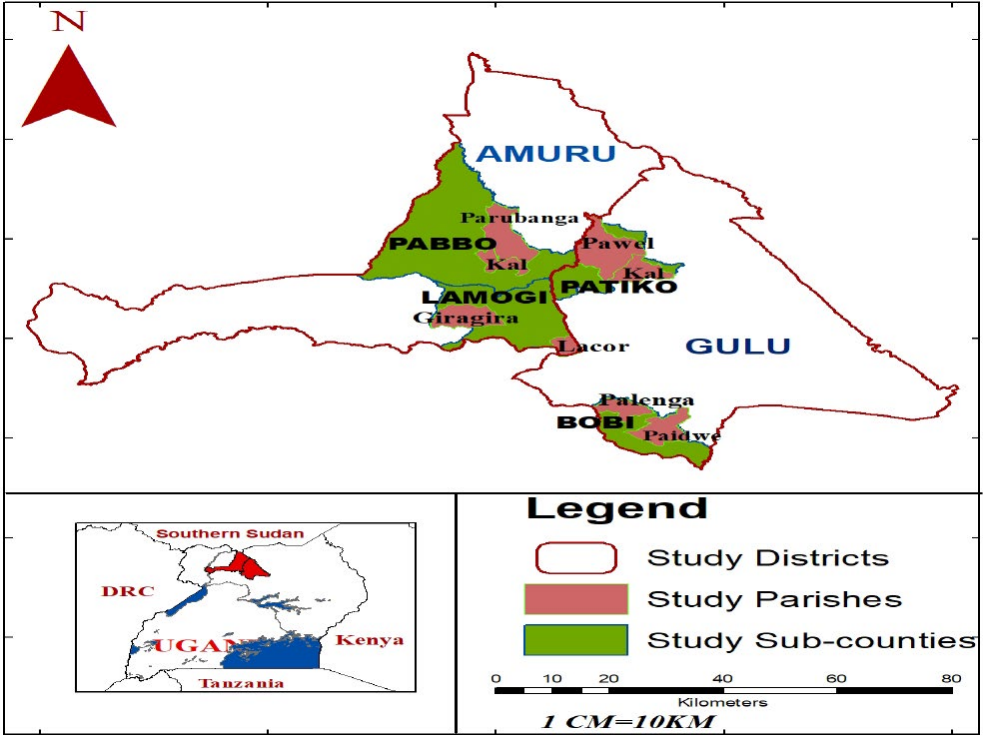
A map showing location of study area

A cross‐sectional study design that made use of primary data from the current study and secondary data from Okidi et al. (2018) was applied. Samples of WFV used for laboratory analyses as well as households that participated in nutritional contribution evaluation were drawn from Amuru and Gulu districts (Figure 1).

Primary data were collected on the level of antinutritional factors and in vitro bioavailability of iron, whereas secondary data on household consumption of WFV were obtained from Okidi et al. (2018). The two districts were purposively selected because previous studies that documented the consumption of WFV were conducted in them (Okidi et al., 2018; Oryema et al., 2013). Second, these two districts are located in Northern region of Uganda where the highest iron deficiency anemia prevalence prevails (UBOS, 2017; Obai et al., 2016; FANTA & USAID, 2010). The two districts cover a total land area of 11,732 km^2^, comprised of open water and swamps (180 km^2^), arable land (10,301 km^2^), national park and game reserves (982 km^2^), and forest covers (371 km^2^) (ACF, 2007).

The main agro ecological zone in the two districts is savannah grassland and characteristically experience both wet and dry seasons (Langdale‐Brown et al., 1964). The average annual rainfall received is approximately 1,500 mm. The wet season extends from April to October with peaks in May, August, and October. The dry season starts in November and lasts up to March (ACF, 2007). The main economic activity in the study area is subsistence agriculture (57.8%). Amuru and Gulu districts have a total population of 634,249 of which 291,457 and 180, 670 live in rural areas of Gulu and Amuru, respectively (Uganda Bureau of Statistics: UBOS, 2014).

### 2.2 *Determination of levels of antinutritional factors and* in vitro *iron bioavailability*

#### 2.2.1 Sample collection and preparation

Samples of 16 wild fruits and vegetables reported to be mostly consumed in Amuru and Gulu districts (Okidi et al., 2018) were collected between October and December 2018 (Table 1).

**Table 1:** Wild fruits and vegetables used in the study (adopted from Okidi et al. (2018))

Wild fruits		Wild vegetables	
Local name	Scientific name	Local name	Scientific name
Oywello	*Vitex doniana* Sweet	Gwanya	*Hibiscus acetosella*
Tugu	*Borassus aethiopum* Mart	Ayuyu	*Acalypha bipartita*
Oceyo	*Aframomum angustifolium* (Sonn.) K.Schum.	Oyado	*Senna obtusifolia*
Cwaa	*Tamarindus indica* L.	Otigo lum/nyim	*Corchorus trilocularis*
Kalara	*Capsicum frutescens* L.	Obuga lum	*Amaranthus spinosus*
Tongogwal Madito	*Physalis macrantha* Link	Pot kalara	*Capsicum frutescens* L.
Kano	*Syzygium malaccense* (L.) Merr. & L.M.Pe	Malakwang Odwonga	NA
		Layika	*Corchorus olitorius*
		Boo ayom/ lok	NA

Note

NA means scientific name is not available.

Three sets of samples were collected randomly from various locations in the two study districts. For each set, approximately 1,500 g of fresh samples of each of the WFV species under investigation was collected. Samples were transported and stored in the refrigerator at 4°C till used. Fruit samples were washed with clean tap water and depulped. The fresh pulps were then oven‐dried at 45°C for 72 hr as described by Okullo et al. (2010) and ground to fine powder using an electric grinder (Brooks Crompton Series 2000, Bradford, UK). For vegetable samples, leaves were picked from stems, washed with tap water, rinsed, oven‐dried, and ground using the same electric grinder already stipulated with cleaning in between samples. Ground samples of each fruit and vegetable species were packed in airtight high‐density poly ethylene (HDPE) for laboratory analyses.

#### 2.2.2 Determination of antinutritional factors

The antinutritional factors considered in this study were phytate, oxalate, tannins, saponin, and total phenols. These antinutritional factors were chosen because of their potential to bind and interfere with bioavailability of iron from plant‐based foods (Natesh et al., 2017). Phytate was determined using high‐performance liquid chromatography (HPLC) procedure previously developed by Lehrfeld (1987). The HPLC equipment used was HPLC‐8100 (Spectra‐physics, San Jose, USA) coupled to SP8440 UV‐vis detector (Beckman instruments, California, USA). For specific separation of phytate, a PRP‐1 5Nm (150 × 4.1 mm) reverse‐phase analytical column was used. The mobile phase consisted of methanol–formic acid solution (1:1.2 ratio). Oxalate content of samples was determined using an enzymatic spectrophotometric method as previously applied by Milardovic *et al*. (2000). The enzymes were obtained from Boehringer (Mannheim, Germany). The spectrophotometer used was Spectrod 2000 (Jena, Mannheim, Germany). Measurements (absorbance) were performed at 340 nm. Tannin was analyzed using the spectrophotometric method that applies the Folin–Denis reagent for color development (Schanderi, 1970). The spectrophotometer used for oxalate detection was also applied except that absorbance measurements were performed at 700 nm. The content of tannin was expressed as a percentage. Saponin was determined using the weight difference method previously used by Obadoni and Ochuko (2002) and as slightly modified by Rout and Basak (2015). All the reagents used (ethanol, n‐butanol, and sodium chloride) were of analytical grade and obtained from BDH (BDH, Kampala, Uganda). Total phenols were determined using the Folin–Ciocalteu spectrophotometric assay method as previously described by Marinova et al. (2005). Measurements of absorbance were performed at 750 nm using the same spectrophotometer model used for oxalate and tannin quantification. All reagents used (Na_2_CO_3_, Folin–Ciocalteu's phenols) were also of analytical grade and obtained from BDH. The total phenolic content was expressed as mg gallic acid equivalents (GAE)/g dry weight. All analyses were performed in duplicates.

#### 2.2.3 *Determination of* in vitro *bioavailability of iron*

Before bioavailability could be assessed, total iron concentration in the samples had to be determined. This was achieved using the flame atomic absorption spectrophotometer (FAAS) (Analytik Jena, Überlingen, Germany) according to Association of Official Analytical Chemists (AOAC) method number 985.35 (AOAC, 2006). Standards for development of standard curve were AAS grade chemicals (Merck, Malaysia), while other reagents (HCl, LiCl) were of analytical grade and obtained from BDH. Absorbance measurements were performed at 450 nm. Following the determination of total iron content, bioavailability of the micronutrient was determined using in vitro dialysability method. This method determines the fraction of dialysable iron following sequential digestion of the sample in simulated gastric and pancreatic medium (Luten et al., 1996). Digestive enzymes consisting of pepsin and pancreatin and bile salts were obtained from Sigma (St Louis, MO, USA). Pepsin solution was prepared by dissolving 16 g of pepsin (P‐7000, from porcine stomach mucosa) in 100 ml of 0.1M HCl. Pancreatin solution containing 4 g of pancreatin (P‐1750, from porcine pancreas) and 25 g of bile extract (B‐8631, porcine) with 1,000 ml of 0.1M NaHCO_3_ were used. Both the pancreatic and gastric processes were performed according to Chiocchetti et al. (2018). The only modification was that for pancreatic digestion, a segment of dialysis tubing (Ø = 20.4 mm; MMCO of 10k Da; Sigma‐Aldrich, Malaysia) containing NaHCO_3_ (an amount equivalent to the moles of NaOH needed for the pancreatic digestion) was used.

### 2.3 Determination of the contribution of bioavailable iron to household iron requirements

To determine the contribution of bioavailable iron from WFV to the annual household iron requirements, a three‐stage process was followed. First, RDAs for the micronutrient for healthy members in the age groups of 7–12 months, 1–3 years, 4–8 years, 9–13 years, 14–18 years, 19–30 years, 31–50 years, 51–70 years, and >70 years segregated by physiological status in terms of pregnancy or lactation (age groups of 14–18 years, 19–30 years, 31–50 years) and sex (male or female) in a given household were aggregated for a day and converted to annual requirement (365 days) as previously reported by Okidi et al. (2018). The RDA reference for the United States of America and Canadian population first published by Institute of Medicine (2001) and later adopted by Brown et al. (2011) was used. This RDA reference has been applied before in other studies conducted in Uganda (Isabirye et al., 2020; Okidi et al., 2018). Second, secondary data on the aggregated annual consumption levels of various WFV species (quantities in grams) by each household that participated in a previous study (Okidi et al., 2018) and primary data on iron content and bioavailability levels determined in the current study were used to derive the quantity of bioavailable iron consumed by each household over a one‐year period. Third, the contribution of bioavailable iron from WFV to the pooled annual household dietary requirement for iron was computed as a fraction (proportion) of the expected pooled annual household RDA for the nutrient.

### 2.4 Data analysis

Data were analyzed using IBM Statistical Package for the Social Sciences (SPSS) software version 22.0. Kolmogorov–Smirnov and Shapiro–Wilk tests were performed to check for normality of data for various aspects. Normality tests revealed that data on levels of antinutritional factors, levels of total iron, and levels of bioavailability of iron were normally distributed. However, data on percentage contribution of WFV to the pooled household annual RDA for the micronutrient were not normally distributed. On this basis, differences in the level of each antinutritional factor, total iron, and bioavailable iron among fruits or vegetables species studied were determined using one‐way analysis of variance (ANOVA). Means were separated using Tukey's honestly significant difference (HSD) test. Pooled antinutritional factor content, total iron, and bioavailable iron between fruits and vegetables were compared using independent sample *t* test. Finally, the contribution of WFV to the pooled annual RDA for iron among households in the study area was estimated by calculating the median of the percentage contribution attained for the sample size of 192 households that were involved in the study. For all statistical analysis, the level of significance was fixed at 5%.

## 3 RESULTS

### 3.1 Levels of antinutritional factors in wild Fruits and Vegetables

Table 2 presents the levels of antinutritional factors in various wild fruits studied. Generally, the contents of antinutritional factors were dependent on fruit species and the specific antinutritional factor in question. All fruits had tannin contents less than 1% and in some cases not detectable, except in *Capsicum frutescens* which was above that level by approximately 0.6%. The contents of phytate in the fruits ranged from 2.36 to 17.26 mg/100 g, with most of them having at least 10 mg/100 g except for *Borassus aethiopum*, *Aframomum angustifolium*, and *Physalis macrantha*.

**Table 2:** Concentration of antinutritional factors in wild fruits

Fruit	Tannins (%)	Phytate (mg/100 g)	Oxalates (%)	Saponin (%)	Total phenolics (mg GAE.g^−1^)
Oywello (*Vitex domiana* Sweet)	0.21 ± 0.03^cb^	17.26 ± 2.52^a^	0.31 ± 0.03^b^	0.27 ± 0.10^cb^	15.60 ± 0.71^c^
Oceyo (*Aframomum angustifolium*/Sonn.)	0.36 ± 0.11^b^	5.18 ± 0.20^c^	1.18 ± 0.06^a^	0.34 ± 0.12^cb^	44.92 ± 1.63^ba^
Kalara (*Capsicum frutescens* L.)	1.59 ± 0.27^a^	10.30 ± 0.86^ba^	0.21 ± 0.04^c^	5.51 ± 1.71^a^	56.41 ± 1.58^a^
Tongongwal Madito (*Physalis macrantha* Link)	0.00 ± 0.00^c^	5.83 ± 0.81^b^	0.00 ± 0.00^d^	1.19 ± 0.26^b^	30.77 ± 0.69^cb^
Kano (*Syzygium malaccense* L./Merr. & L.M.Pe)	0.26 ± 0.08^a^	17.26 ± 7.11^a^	0.00 ± 0.00^d^	0.02 ± 0.00^cb^	52.20 ± 1.18^ba^
Cwa (*Tamarindus indica* L.)	0.04 ± 0.02^c^	15.41 ± 1.46^a^	0.04 ± 0.01^d^	0.03 ± 0.01^cb^	60.30 ± 1.45^a^
Tugu (*Borassus aethiopum* Mart)	0.00 ± 0.00^c^	2.36 ± 0.00^c^	0.00 ± 0.00^d^	0.00 ± 0.00^c^	9.93 ± 1.65^c^

Note

Values indicate mean ± *SD* (*n* = 6). Each plant material was collected from three different locations and analyzed in duplicates. Values with different superscripts in the same column are significantly different (*p* < .05).

There was some clustering of fruit species in terms of the levels of the antinutritional factors. The levels of phytates were the same in *Vitex doniana, Syzygium malaccense*, and *Tamarindus indica* (cluster 1), *Physalis macrantha and Capsicum frutescens* (cluster 2), and *Borassus aethiopum* and *Aframomum angustifolium* (cluster 3). Among all the fruits examined, oxalates were detected only in *Aframomum angustifolium*, *Vitex doniana*, and *Capsicum frutescens*. In species where detection occurred, the concentration of oxalate ranged from 0.21% to 1.18%, with the highest concentration found in *Aframomum angustifolium*, followed by *Vitex doniana*, and least in *Capsicum frutescens* in decreasing order of magnitude. Saponin content of all the fruits examined was less than 1% except in *Capsicum frutescens* and *Physalis macrantha* which had levels above one but less than 7%. The total phenolic content of the fruits ranged from 9.93 to 60.30 mg GAE.g^−1^. However, most (71%) of them had total phenolic concentration above 30 mg GAE.g^−1^. The highest total phenolic content was detected in *Tamarindus indica*, followed by *Capsicum frutescens*, and *Syzygium malaccense* in decreasing order of magnitude.

The concentrations of antinutritional factors in wild vegetables investigated are presented in Table 3. As was observed for wild fruits, in this case, the contents of antinutritional factors also varied across the vegetable species and specific antinutritional factors. For antinutritional factors whose contents were expressed in percentage points (tannins, oxalates, and saponin), their levels in the plants were generally less than 1% and were observed in 78, 89, and 56% of the vegetables examined for tannins, oxalates, and saponin, respectively. Among the three antinutritional factors, saponin was the most widely distributed and the highest concentration was detected in *Amaranthus spinosus* at a level of about 10 times above other vegetable species. Phytate was detected in all the vegetable species examined with concentration ranging from 75.7 to 227 mg/100 g depending on the vegetable species. At least half of the vegetable species examined had phytate at concentration above 150 mg/100 g. For those vegetable species that had phytate concentration above 150 mg/100 g, the highest concentration was detected in *Corchorus trilocularis* (227 mg/100 g), *Hibiscus acetosella* (218.52 mg/100 g), *Corchorus olitorius* (217.45 mg/100 g), *Acalypha bipartita* (188.45 mg/100 g), and Malakwang odwonga (173.62 mg/100 g) in decreasing order of magnitude. Total phenolics were also detected in all the vegetable species examined. The levels varied with the vegetable species and ranged from 14 to 66 mg GAE.g^−1^.

**Table 3:** Concentration of antinutritional factors in wild vegetables

Vegetables	Tannins (%)	Phytate (mg/100g)	Oxalates (%)	Saponin (%)	Total phenolics (mg GAE.g^−1^)
Oyado (*Senna obtusifolia*)	4.28 ± 0.43^a^	83.30 ± 2.30^c^	0.18 ± 0.03^b^	2.11 ± 0.27^b^	38.19 ± 1.86^cd^
Pot kalara (*Capsicum frutescens* L.)	1.53 ± 0.10^b^	75.66 ± 1.56^c^	0.22 ± 0.02^b^	1.58 ± 0.37^b^	31.58 ± 2.81^d^
Otigo lum/nyim (*Corchorus trilocularis*)	0.39 ± 0.03^c^	226.96 ± 2.60^a^	0.02 ± 0.00^c^	0.38 ± 0.06^b^	65.76 ± 1.91^a^
Ayuyu (*Acalypha bipartita*)	0.23 ± 0.01^c^	188.45 ± 4.62^ab^	0.13 ± 0.00^bc^	0.23 ± 0.06^b^	58.07 ± 1.54^ab^
Gwanya (*Hibiscus acetosella*)	0.14 ± 0.03^c^	218.52 ± 4.45^a^	0.20 ± 0.03^b^	1.98 ± 0.51^b^	44.71 ± 0.87^bcd^
Obuga lum (*Amaranthus spinosus*)	0.05 ± 3.23^c^	94.06 ± 2.16^c^	1.08 ± 0.17^a^	15.55 ± 0.91^a^	40.06 ± 2.81^cd^
Layika (*Corchorus olitorius*)	0.02 ± 0.00^c^	217.45 ± 6.00^a^	0.01 ± 0.00^c^	0.10 ± 0.08^b^	52.05 ± 1.57^abc^
Boo ayom/ lok (NA)	0.00 ± 0.00^c^	79.67 ± 1.72^c^	0.12 ± 0.02^bc^	0.00 ± 00^b^	14.38 ± 0.55^e^
Malakwang Odwonga (NA)	0.00 ± 0.00^c^	173.62 ± 2.70^b^	0.15 ± 0.04^bc^	0.00 ± 00^b^	30.40 ± 0.68^d^

Note

Values indicate mean ± *SD* (*n* = 6). Each plant material was collected from three different locations and analyzed in duplicates. Values with different superscripts in the same column are significantly different (*p* < .05). NA means scientific name is not available.

Comparison of pooled concentration of specific antinutritional factors between wild fruits and vegetables is presented in Table 4. Generally, the levels of all antinutritional factors were not different between fruits and vegetables except phytate which was higher in vegetables than fruits by a factor of 14.

**Table 4:** Comparison of pooled concentration of antinutritional factors between wild fruits and vegetables

Antinutritional factors	Fruits	Vegetables	*p*‐value
Tannin (%)	0.35 ± 0.03	0.74 ± 0.04	.09
Phytate (mg/100 g)	10.51 ± 2.46	150.86 ± 6.27	.00
Oxalate (%)	0.25 ± 0.04	0.23 ± 0.01	.85
Saponin (%)	1.05 ± 0.04	2.43 ± 0.19	.09
Total phenolics (mg GAE.g^−1^)	38.59 ± 2.08	41.69 ± 1.50	.43

Note

Values indicate mean ± *SD* (*n* = 54 for vegetables, *n* = 42 for fruits).

### 3.2 Bioavailability of Iron from wild Fruits and Vegetables

With regard to fruits, generally, the concentration of total iron varied with the plant species and ranged from 0.81 to 5.97 mg/100 g (Table 5). All fruits except those from *Borassus aethiopum* and *Physalis macrantha* had iron content above 2 mg/100 g. Specifically, *Capsicum frutescens* and *Vitex doniana* had the highest level of iron, followed by *Aframomum angustifolium*, *Tamarindus indica*, *Syzygium malaccense*, *Physalis macrantha*, and *Borassus aethiopum* in decreasing order of magnitude. Bioavailability of iron from fruits was also dependent on the plant species and ranged from 2.7% to 23%. The degree of bioavailability was not corresponding to the level of total iron in the fruit but randomly distributed. Nevertheless, bioavailability was highest in fruits of *Capsicum frutescens* (22.91%), followed by *Vitex doniana* (17.93%), *Syzygium malaccense* (15.61%), and *Aframomum angustifolium* (15.14%), while the rest had bioavailability levels below 5%.

**Table 5:** Concentration and bioavailability status of iron from wild fruits and vegetables

Wild fruits			Wild vegetables		
	Total iron (mg/100 g)	Bioavailability (%)		Total iron (mg/100 g)	Bioavailability (%)
Oywello (*Vitex doniana* Sweet)	5.16 ± 0.61^a^	17.93 ± 3.14^b^	Gwanya (*Hibiscus acetosella*)	0.49 ± 0.04^c^	9.80 ± 0.530^ed^
Oceyo (*Aframomum angustifolium*/Sonn.)	3.88 ± 0.40^b^	15.14 ± 2.81^b^	Obuga lum (*Amaranthus spinosus*)	1.06 ± 0.03^c^	22.65 ± 3.23^b^
Kalara (*Capsicum frutescens* L.)	5.97 ± 1.01^a^	22.91 ± 2.49^a^	Oyado (*Senna obtusifolia*)	2.35 ± 0.40^b^	10.15 ± 1.43^ed^
Tongogwal Madito (*Physalis macrantha* Link)	0.99 ± 0.08^d^	2.65 ± 0.08^c^	Pot kalara (*Capsicum frutescens* L.)	0.25 ± 0.01^c^	6.15 ± 1.10^e^
Kano (*Syzygium malaccense L*./Merr. & L.M.Pe)	2.15 ± 0.16^c^	15.61 ± 3.02^b^	Otigo lum/nyim (*Corchorus trilocularis*)	5.67 ± 1.25^a^	18.37 ± 0.80^cb^
Cwa (*Tamarindus indica* L.)	2.99 ± 0.13^cb^	4.09 ± 0.11^c^	Malakwang Odwonga (NA)	0.26 ± 0.01^c^	12.36 ± 1.15^d^
Tugu (*Borassus aethiopum Mart*)	0.81 ± 0.01^d^	3.56 ± 0.68^c^	Boo ayom (NA)	0.25 ± 0.02^c^	12.51 ± 2.16^d^
			Layika (*Corchorus olitorius*)	0.94 ± 0.01^c^	27.65 ± 1.72^a^
			Ayuyu (*Acalypha bipartita*)	0.25 ± 0.02^c^	13.83 ± 0.93^cd^

Note

Values indicate mean ± *SD* (*n* = 6). Each plant material was collected from three different locations and analyzed in duplicates. Values with different superscripts in the same column are significantly different (*p* < .05). NA means scientific name is not available.

In the case of vegetables, iron was detected in all the vegetable species investigated (Table 5). As was the case for fruits, iron content in vegetables was also dependent on plant species and ranged from 0.25 to 5.7 mg/100 g. Iron content was generally below 1% and at the same level on average (*p* > .05) except for *Corchorus trilocularis*, *Senna obtusifolia*, and *Amaranthus spinosus*. In situation where iron content was above 1%, the highest concentration was detected in *Corchorus trilocularis*, followed by *Senna obtusifolia*, and *Amaranthus spinosus* in decreasing order of magnitude. Iron bioavailability among vegetables varied between 6.15% and 27.65%. Iron bioavailability among vegetables was highest and least in *Corchorus olitorius* and *Capsicum frutescens*, respectively. Estimated 78% of the vegetables studied contained biologically available iron above 10%. As observed in the case of fruits, the degree of bioavailability of iron was not concomitant with the level of total iron. Pooled concentration of total iron and iron bioavailability in fruits or vegetables is presented in Table 6. Generally, iron was more abundant in fruits than in vegetables by a factor of about 2 (*p* < .05). However, iron was more bioavailable in vegetables than in fruits by approximately 3.2% (*p* < .05).

**Table 6:** Pooled concentration and bioavailability of iron from wild fruits and vegetables

Parameter	Fruits	Vegetables	*p*‐value
Total iron (mg/100 g)	3.14 ± 0.15	1.30 ± 0.04	.00
Iron bioavailability (%)	11.69 ± 1.03	14.86 ± 1.88	.04

Note

Values indicate mean ± *SD* (*n* = 42 for fruits, *n* = 54 for vegetables).

### 3.3 Contribution of bioavailable iron by wild fruits and vegetables to household iron requirements

**Figure 2:**
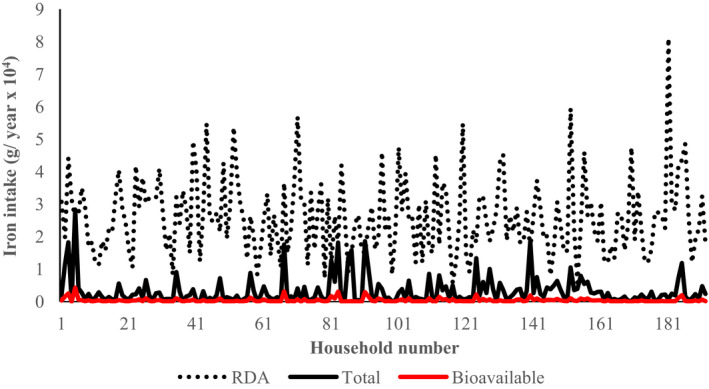
Total and biologically available iron derived from wild fruits. RDA indicates the annual pooled value for the household from members aged 7–12 months, 1–3 years, 4–8 years, 9–13 years, 14–18 years, 19–30 years, 31–50 years, 51–70 years, and >70 years

The distribution of the level of intake of total iron, bioavailable iron from wild fruits against the expected annual pooled household RDA for iron as stipulated under section 2.3 among households (192) that participated in the study is presented in Figure 2.

Generally, for most of the households, intake of total and bioavailable iron from wild fruits was all below the pooled annual household RDA. In terms of total iron, the median contribution of wild fruits to the pooled annual household RDA was 7.6% (a minimum of 0.04 and a maximum of 118%). On the other hand, in terms of bioavailable iron, wild fruits had a median contribution of 0.8% (a minimum of 0.00 and a maximum of 19.5%) to the pooled annual RDA among households. Approximately 3.6% of the households had the contribution from wild fruits at or above the pooled annual RDA for iron on the basis of total iron. However, none of the households had contribution from wild fruits matching the pooled annual RDA for the micronutrient in terms of the bioavailable fraction.

**Figure 3:**
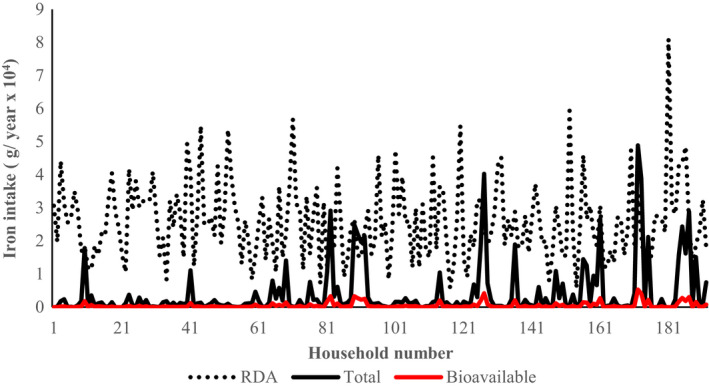
Total and biologically available iron derived from wild vegetables. RDA indicates the annual pooled value for the household from members aged 7–12 months, 1–3 years, 4–8 years, 9–13 years, 14–18 years, 19–30 years, 31–50 years, 51–70 years, and >70 years

Distribution of the level of total and bioavailable iron from wild vegetables matched against the expected pooled annual RDA among households (192) that participated in the study is presented in Figure 3. As was observed in the case of fruits, for the case of vegetables, majority of households had the pooled annual RDA above total and bioavailable iron derived from wild vegetables.

Generally, in terms of total iron intake, 4.3% of the households had the contribution from wild vegetables matching the pooled annual RDA for the micronutrient. The wild vegetables studied had a median contribution of 4.6% (a minimum of 0.05 and a maximum of 144%) to the pooled annual RDA among households on the basis of total iron. In terms of bioavailable iron, a median contribution of 0.60% (minimum of 0.00 and a maximum of 18.8%) to the pooled annual RDA was achieved among households through consumption of wild vegetables. However, none of the households had contribution from wild vegetables matching the pooled annual RDA for iron in terms of the bioavailable fraction.

**Figure 4:**
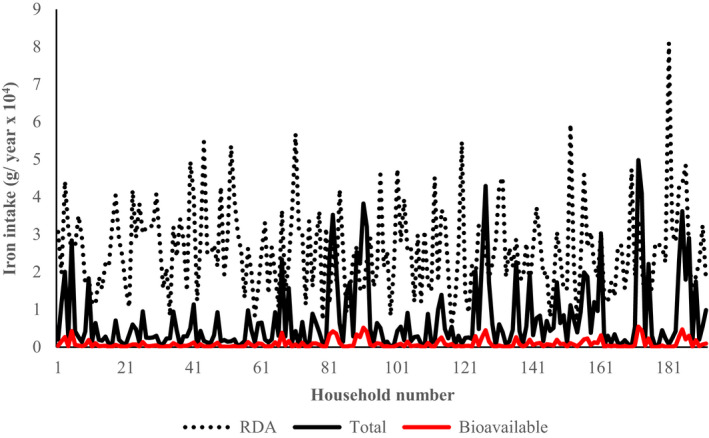
Total and biologically available iron derived from wild fruits and vegetables. RDA indicates the annual pooled value for the household from members aged 7–12 months, 1–3 years, 4–8 years, 9–13 years, 14–18 years, 19–30 years, 31–50 years, 51–70 years, and >70 years

The distribution of the level of pooled intake of total and bioavailable iron from WFV in comparison to the annual pooled RDA among households (192) that participated in the study is presented in Figure 4. In general, majority of the households (91%) had total iron intake from WFV below the pooled annual RDA, while none of the households had contribution from the same matching the pooled annual RDA in terms of bioavailable iron. Nonetheless, WFV had a median contribution of 14.9% (a minimum of 0.11 and a maximum of 150.2%) and 1.8% (a minimum of 0.02 and a maximum of 34.7%) to the pooled annual RDA for total and bioavailable iron, respectively, among households.

## 4 DISCUSSION

In the current study, the levels of antinutritional factors in WFV commonly consumed in Acholi subregion of Uganda were examined (Table 1). It is apparent that the concentration of the antinutritional factors varied with plant species as well as the antinutritional factor under consideration (Table 2 and 3). This observation is not peculiar to this study but has also been reported in previous studies conducted elsewhere (Agbaire & Emoyan, 2012; Rout & Basak, 2015). Comparing the results of this study with the findings from other studies reveals two interesting scenarios. First, the tannin, oxalate, and saponin contents of the wild food plants investigated being less than 1% as revealed by the study concurs with the findings on wild food plants reported in previous studies conducted in Kolhapur and Odisha districts of India and Benue, Oyo, Osum, and Abeokuta states of Nigeria, respectively (Rathod & Valvi, 2011; Rout & Basak, 2015; Anhwange et al., 2015; Ajala, 2009; Bello et al., 2008; Adeboye & Babajide, 2007). Second, there is a contrasting scenario with respect to the content of phytate and oxalate in the sense that while Rout and Basak (2015) reported similar tannin and saponin content as is the case with the present study, the content of both phytate and oxalate was greater than the levels reported in the present study. Similarity between the results of the current study and that of Rout and Basak (2015) with respect to particular antinutritional factors but not all of them can be attributed to random variation of plant components among different plant species (Chattoo et al., 2011; Schuldt et al., 2019). This fact can be justified by the work of Rathod and Valvi (2011) which showed similar tannin and oxalate contents but not saponin content with that recorded in the present study.

The levels of oxalate, tannin, and saponin in WFV species detected in the current study are comparable to those reported for conventional plant species such as Pumpkins (*Cucurbita spp*.), Spinach (*Spinacia oleracea*), Okra (*Abelmoschus esculentus*), and Chick peas (*Cicer arietinum*) (Nwogwugwu et al., 2016; Sinha & Khare, 2017). This observation challenges the common belief that WFV contain higher contents of antinutritional factors than the domesticated variants (Natesh et al., 2017). Comparing the pooled content of each antinutritional factor between fruits and vegetables indicates identical levels except phytate which was significantly higher in vegetables than in fruits (Table 4). This suggests that wild vegetables would provide less bioavailable iron due to the high content of the phytate, a principal antinutritional factor that binds iron (Natesh et al., 2017). A critical look at individual wild vegetables studied revealed that *Corchorus trilocularis*, *Hibiscus acetosella*, *Corchorus olitorius*, *Acalypha bipartita*, and Malakwang Odwonga had much higher content of phytate. This suggests that diets consisting of them would dramatically reduce iron utilization.

Bioavailability is one of the critical factors important in ascertaining nutrient intake adequacy from a given diet (Delimont et al., 2017; Rousseau et al., 2019). Whereas wild food plants are reported to be essential for micronutrient nutrition among disadvantaged communities in developing countries, understanding the status of bioavailability of micronutrients from wild food plant resources such as those consumed in Acholi subregion of Uganda is necessary to provide indications on what households derive from them. With specific reference to iron, competitive nutrient absorption studies indicate that several other nonessential metals (e.g., Pb^2+^ and Cd^2+^) share iron intestinal absorption pathway, a factor which makes understanding its availability for absorption critical (Chijioke et al., 2019; Meltzer et al., 2010).

The results of this study have revealed that bioavailability of iron was dependent on plant species (Table 5). This variation can be attributed to differences in the composition of antinutritional factors and their respective levels in various plant species investigated (Table 2 and 3). Average iron absorption from heme food sources such as meat range from 15% to 35%. However, it varies from 40% during iron deficiency to 10% during iron repletion (Hurrell & Egli, 2010). On the other hand, for plant‐based diets iron bioavailability can be as low as 5%–12% (Hurrell & Egli, 2010; Blanco‐Rojo & Vanquero, 2018). This low level of iron bioavailability from plant‐based diets expose resource constrained households especially those in rural areas of developing countries to persistent iron deficiency and related complications.

There is sufficient evidence to suggest that antinutritional factors in plant‐based foods contribute to low iron bioavailability (Biesalski & Black, 2016; Natesh et al., 2017; Welch & Graham, 2009). Among antinutritional factors that lower iron bioavailability, the most cited include tannins, phytate, oxalate, saponin, and total phenolics that were all considered in the present study (Table 2). The present study recorded iron bioavailability of 11.7% from fruits and 14.8% from vegetables (Table 6). These values are supported by the work of Scheers et al. (2015), but lower than the values reported by Chiocchetti et al. (2018) for pumpkin peels (20% bioavailability). The lower bioavailability observed in the present study can be attributed to higher antinutritional factors content especially phytate and total phenolics which were greater in plant species used in the present study (Table 2 and 3) than reported in previous studies (Banjari et al., 2013; Ajala, 2009; Ndlovu & Afolayan, 2008; Adeboye & Babajide, 2007; Castro‐ Alba et al., 2019). This further justifies the rationale for the current study thus signifying limited application of literature information when evaluating nutritional efficacy of wild food plants among studies conducted in different geographical locations.

Other nutritional‐related studies have revealed that addition of certain fruits or vegetables containing ascorbic acid to a meal may double or even triple the absorption of iron depending on the amount of ascorbic acid present (Wanling et al., 2019). This suggests that beside the levels of antinutritional factors in the studied fruits and vegetables, the levels of ascorbic acid in individual fruit and/or vegetable may have contributed to the variation in iron bioavailability. However, ascorbic acid levels were not considered in the present study. Future studies should consider ascorbic acid levels in wild fruits and vegetables to better understand the contribution of antinutritional factors alone to bioavailability of iron.

On the other hand, it has been shown that bioavailability of nonheme iron (in terms of absorption) such as those found in plant foods increases in the presence of heme iron (Kumar et al., 2020; Young et al., 2018). This implies that consumption of food groups that contain heme iron such as meat together with WFV could potentially increase bioavailability of iron from them. However, an important question that has largely remained unanswered is the quantity of heme iron food group that is required to substantially improve bioavailability of nonheme iron from plant foods. This question is important considering the fact that heme iron food sources such as meat are rather expensive and are only limitedly affordable by economically disadvantaged households that are major consumers of wild food resources (Asprilla‐Perea & Diaz‐Puente, 2019; Zulu et al., 2019).

It was noted that whereas wild fruits had more iron than the vegetable species, iron bioavailability was higher in vegetables than in fruits (Table 6). This observation is not peculiar to the current study. Previous studies have shown that bioavailability of iron decreased with increase in the concentration of iron in plant foods (Hurrel & Egli, 2010) ostensibly due to higher contents of antinutritional factors (Natesh et al., 2017; Acipa *et al*., 2013). From a nutritional point of view, nutrients in any food source are useful to the body if they can be utilized by the cells and tissues to support body functions (Moughan, 2018). Whereas nutrient bioavailability is a proximate indicator of the nutritional value of a food source to the human body (Baree *et al*., 2018), knowledge of bioavailability alone may not be adequate to construe nutrient adequacy at household level, unless meaningfully translated to reflect RDA of household members. Indeed, in this study, the fraction of total iron in WFV available for metabolism over a one‐year period was assessed. Contrary to the general belief that wild food plants are essential to households’ nutrition among rural households in developing country setting such as the Acholi subregion of Uganda (Okidi et al., 2018), the contribution of bioavailable iron to household annual RDA was very marginal (Figure 4). This indicates that wild food plants cannot be relied upon to support iron needs of households in a developing country setting such as the Acholi subregion of Uganda. Despite this limitation, it is important to appreciate that wild food plants contain other nutrients such as vitamin A (in the form of provitamin, β‐carotene) for which bioavailability is not constrained by antinutritional factors. Therefore, the limited nutritional value of WFV in terms of iron should not limit households from consuming them.

From a nutritional point of view, it is well known that no food type contains all nutrients in adequate quantities but nutrient complementarity can be achieved through diet composites derived from various food sources. This is indeed the fundamental basis for emphasis on dietary diversity (Alowo et al., 2018). Therefore, households should be encouraged to include other high iron‐bioavailable food sources such as meat in the diet. Relatedly, whereas dietary diversity is believed to be a good indicator of diet adequacy (Alowo et al., 2018), information on optimal combination of food types to ensure diet adequacy for micronutrients of public health importance such as iron, vitamin A, and zinc for use by economically disadvantaged households in developing countries such as those in Acholi subregion of Uganda is largely lacking. This is a potential subject for future research.

In looking at these results, it should be appreciated that calculation of the contribution of bioavailable iron from WFV to household RDA did not take into account processing methods (practiced in Acholi subregion such as sun drying, boiling, and sun drying, and salting and sun drying) that can improve bioavailability of iron from plant foods (Bighaghire, 2019). Thus, the abysmal contribution observed in the current study may be an underestimation. Future studies should evaluate the effect of those processing methods on iron bioavailability from WFV. When segregated botanically, the contribution of bioavailable iron to household RDA was below 2% for both fruits and vegetables although contribution in terms of total iron intake was more for fruits than vegetables by a difference of 13.3%. This observation is not surprising because iron from fruits was less bioavailable than from vegetables (Table 6).

Generally, majority of rural households such as those living in Acholi subregion of Uganda depend mostly on plant‐based foods for which bioavailability of iron and other essential micronutrients such as zinc is largely constrained by the presence of antinutritional factors (Biesalski & Black, 2016). Okidi et al. (2018) reported that WFV contributed adequately to household iron requirement in Acholi subregion on the basis of total iron content of the wild food plants. An important argument that formed the basis of the current study was that contribution based on total iron content of wild food plants would be an overestimation because plant foods contain antinutritional factors that reduce bioavailability of iron. Indeed, at least at in vitro scale the results concur with the initial argument. On the other hand, the level of contribution based on total iron content of the plant foods investigated is far much lower than what was reported by Okidi et al. (2018). The differences could be attributed to the fact that Okidi et al. (2018) used literature values for iron, disregarding the fact that iron content of foods is critically dependent on geographical location with varying soil and climatic conditions (Abdelgawad et al., 2014; Kumar & Sangwan, 2019; Uusiku et al., 2010). This indicates that literature values did not reflect real iron contents of the WFV in Acholi subregion of Uganda.

On the other hand, it should be noted that fruits and vegetables also contain bioactive compounds that are essential for human health. The health promoting effects of fruits and vegetables are believed to be due to two main factors: (a) their contents of natural dietary phytochemicals (Liu, 2013) which can potentially reduce the chances of occurrence of noncommunicable diseases such as hypertension and diabetes (Tsuda, 2016; Marrelli et al., 2014; Koss‐Mikołajczyk et al., 2019) and (b) their high content of water and low metabolizable energy levels which can retard the development of conditions that exacerbate the occurrence of noncommunicable diseases such as high body weight and high blood sugar accumulation (Marrelli et al., 2014; Koss‐Mikołajczyk et al., 2019). Thus, because of these health benefits, the World Health Organization recommends daily consumption of at least 400 g of fruits and vegetables or five servings per day (FAO & WHO, 2004). Therefore, despite the abysmal contribution of wild fruits and vegetables to household iron needs, their consumption should be encouraged because of the afore‐stated health benefits. This is particularly important during lean seasons when other cultivated plant food sources are only limitedly available (Aberoumand & Deokule, 2009; Agea et al., 2011).

This study encountered two limitations. First, nutrient interactions are one of the factors that interfere with iron bioavailability. However, this study did not assess the effect of nutrient interaction on iron bioavailability. Future studies should design methods that allow examination of the contribution of antinutritional factors and other nutrients to iron bioavailability separately. Second, this study used secondary data built on the assumption that food distribution in households is in accordance with individual household member food needs which may not be the case. Therefore, nutrient adequacy determined based on pooled household estimate may not reflect the intrahousehold nutrient adequacy.

## 5 CONCLUSIONS

This study has demonstrated that the pool of antinutritional factors in wild food plants in Acholi subregion is dominated by phytate and total phenolics, while bioavailability of iron is higher from wild vegetables than from wild fruits despite the later having higher content of iron than the former. Taking into consideration the limitations of this study, the contribution of bioavailable iron to household annual requirement for the micronutrient was very marginal. Thus, wild fruits and vegetables alone cannot be relied upon to guarantee adequate intake of iron among rural households in Acholi subregion of Uganda. Future studies should consider establishing optimal food combinations that can enhance uptake of iron from WFV, the effect of bioenhancers such as vitamin C and nutrient interaction on bioavailability of iron from wild food plants as well as the effect of traditional processing methods on levels of antinutritional factors, bioavailability, and annual intake of bioavailable iron at individual household member level.

## CONFLICT OF INTEREST

The authors declare no conflict of interest.

## ETHICAL APPROVAL

Ethical clearance was obtained from Gulu University Research Ethics Committee (Approval number: GUREC‐079–18). Permission from Chief Administrative Officers was also sought before data collection in the respective districts. Last but not least, informed consent of residents who helped in the identification and collection of study samples was obtained prior to sample collection.

## Data Availability

Data that support the findings of this study are available from the corresponding author upon reasonable request.
